# Elevated Concentrations of Soluble Fas and FasL in Multiple Sclerosis Patients with Antinuclear Antibodies

**DOI:** 10.3390/jcm9123845

**Published:** 2020-11-26

**Authors:** Josip Sremec, Sanja Tomasović, Nada Tomić Sremec, Alan Šućur, Jelena Košćak Lukač, Koraljka Bačić Baronica, Danka Grčević, Nataša Kovačić

**Affiliations:** 1 Department of Neurology, “Sveti Duh” Clinical Hospital, Sveti Duh 64, 10000 Zagreb, Croatia; jkoscak47@gmail.com; 2 Neurology Clinic, Faculty of Medicine, Josip Juraj Strossmayer University of Osijek, Sveti Duh 64, 10000 Zagreb, Croatia; stomasovic98@gmail.com (S.T.); koraljka7@yahoo.com (K.B.B.); 3 Division for Laboratory Immunology, Department of Laboratory Diagnostics, University Hospital Center Zagreb, Kišpatićeva 12, 10000 Zagreb, Croatia; nada-tomic@hotmail.com; 4 Department of Physiology and Immunology, University of Zagreb School of Medicine, Šalata 3, 10000 Zagreb, Croatia; alan.sucur@mef.hr (A.Š.); danka.grcevic@mef.hr (D.G.); 5 Department of Anatomy, University of Zagreb School of Medicine, Šalata 11, 10000 Zagreb, Croatia; natasa.kovacic@mef.hr

**Keywords:** multiple sclerosis, antinuclear antibodies, sFas, sFasL, autoimmunity, apoptosis

## Abstract

Antinuclear antibodies (ANA) are currently considered as an epiphenomenon of apoptotic processes, possibly in control of autoreactivity in patients with multiple sclerosis (MS). Apoptosis of reactive lymphocytes by the Fas/FasL system is described as an effective control mechanism for autoreactivity in MS. We aimed to provide a context to the potential link between ANA and peripheral lymphocyte apoptosis in MS. The presence of ANA was detected in sera by immunofluorescence assay, and concentrations of sFas and sFasL were determined in the sera of 44 and cerebrospinal fluid (CSF) of 11 relapsing-remitting (RR) MS patients using cytometric bead-based array, and their association with the disease characteristics was determined. ANA were detected in the sera of 43.2% of RRMS patients, and their frequency was the highest in patients with disease duration of less than one year (88,89%). In addition, the number of experienced relapses was lower in ANA-positive patients. Concentrations of sFasL were inversely associated with patients’ expanded disability status scale (EDSS) scores. Low concentrations of both soluble factors strongly discriminated patients with moderate to severe disability, from patients with mild or absent disability only in a group of patients with prolonged disease duration (>10 years). Both soluble mediators were significantly higher in ANA-positive patients. FasL concentrations were inversely associated with the number of relapses. There is a potential link between the presence of ANA and peripheral lymphocyte apoptosis mediated by Fas/FasL system in MS, whose precise role and significance needs to be determined by future mechanistic studies.

## 1. Introduction

Multiple sclerosis (MS) is a chronic, demyelinating immune-mediated disorder [1] characterized by multiple alterations in various components of the immune system. The complexity of the pathogenic mechanisms underlying the disease and associated diagnostic, prognostic, and therapeutic challenges resulted in the multitude of diagnostic or prognostic biomarkers, which include genetic variants (HLA-DRB1 polymorphisms), immune-related factors (oligoclonal bands, reactivity to viruses, auto-antibodies to myelin components), and metabolically active neural homeostatic molecules (vitamin D) [2,3,4,5,6,7].

Among autoantibodies, antinuclear antibodies (ANA), which are frequently found in systemic autoimmune rheumatic diseases (SARD), such as systemic lupus erythematosus (SLE), Sjögren’s syndrome, and systemic sclerosis, have also been recognized in higher frequencies in MS patients than in healthy individuals [8,9,10]. Earlier studies considered ANA positivity in MS as a false positive finding [11], or recommended their exclusion during evaluation of clinically isolated syndrome (CIS) and MS, argumenting that their presence in the context of MS was not indicative of an alternative diagnosis, such as a disease from the SARD spectrum [12]. Others reported the association of ANA positivity with the activity of MS [13], or pointed out their intermittent presence over the course of MS [14]. There are also reports on the development of signs of SARD diseases in only a small fraction of ANA positive patients with CIS [15]. Recent study described the existence of CNS-specific ANA, associated with greater disability and reflecting the transition to the progressive phase of the disease [16]. ANA positivity is also commonly detected in neuromyelitis optica disease spectrum (NMOSD), which partially corresponds to MS [17,18], and seems to be associated with milder disease course and fewer relapses [19].

The mechanisms underlying the association of ANA with the pathogenesis of MS is still not completely understood, in contrast to the pathogenesis of SARD, which more clearly involves ANA-mediated tissue damage. Direct pathogenic role of ANA is confirmed only in SLE, where anti-dsDNA (double-stranded DNA) antibodies are directly responsible for tissue damage [20]. Research carried out in other systemic autoimmune diseases indicates that the presence of ANA might not reflect their direct pathogenic role, and instead could be a consequence of defective, enhanced, or prolonged apoptotic processes in the organism, resulting in increased exposure of cellular nuclear antigens to the immune system, and subsequent production of autoantibodies [21,22]. Frequent presence of ANA in patients with MS could thus be ascribed to altered apoptotic processes in the CNS, or in the periphery.

One of the main apoptotic pathways involved in the regulation of immune response is the Fas/Fas ligand system. Fas (APO-1, CD95) is a membrane receptor belonging to the tumor necrosis factor (TNF) superfamily [23]. Binding of its membrane-bound ligand (Fas ligand, FasL, CD95L, CD178), transmits the apoptotic signal through the activation of intracellular cysteine-dependent aspartate-specific proteases (caspases) [24]. Fas is expressed on various normal and malignant cells, making them susceptible to damage by the immune system [25,26], whereas expression of FasL is restricted to cells of the immune system [27], and immunologically privileged sites, such as testis, eye, or the placenta [28]. The Fas/FasL system has an important role in restricting the lymphocyte activation, via mechanisms such as activation induced cell death (AICD) [25]. In addition to membrane-bound forms, there are soluble forms of Fas and FasL. Soluble (s)Fas is generated through alternative mRNA processing [29], and competitively blocks Fas activation by FasL [30]. Binding of sFasL to Fas expressed on T-lymphocytes prevents the binding of membrane bound form and thus protects them from apoptosis. However, sFasL binding could also result in non-apoptotic Fas-signaling implicated in proinflammatory activity of Fas [31].

The role of apoptotic processes in MS has been well documented, both in the central nervous system (CNS), where it pertains to apoptosis of oligodendrocytes, and in the periphery, where those processes are responsible for the removal of autoreactive lymphocytes [32]. It is already confirmed that the Fas/FasL system presents an important mechanism controlling the T-cell activity in MS [27]. Increased expression of Fas and FasL mRNA in peripheral blood mononuclear cells from MS patients is associated with a slower progression of the disease [33]. Increased concentrations of sFas have been reported in the sera of MS patients during relapse [34].

The aim of this study was to analyze the presence of ANA and the concentration of soluble components of the Fas/FasL system, and their association with disease characteristics, as well as their mutual association in patients with RRMS.

## 2. Materials and Methods

The study included 44 patients with relapsing-remitting MS (RRMS) (32 female, 12 male), defined by the revised McDonald’s criteria [35], recruited during their stay in “Sveti Duh” Clinical hospital. All patients were within 15 days of a clinical relapse. Samples were obtained either before the initiation of corticosteroid treatment, or within the same day after their initiation. Due to their delayed onset of effects [36], it is not likely that treatment would affect the inflammatory or apoptotic processes within the first day. Patients not currently in a relapse, patients with confirmed diagnosis or clinical suspicion of SARD, patients currently or previously taking DMT therapy, and patients who have undergone immune reconstitution therapy have been excluded from the study. It was possible to accumulate a sufficient number of treatment naïve patients because the national healthcare insurance company provided reimbursement for DMT therapy only for a small fraction of MS patients with aggressive disease at the time of the enrollment process. The participants had no history of other illnesses that could modulate the apoptotic processes, including all forms of acute or chronic inflammatory (especially autoimmune) diseases, malignant tumors, or infections.

The patients have been recruited after obtaining institutional ethics approval number 01-2994/13 issued by ethics committee of the Clinical hospital “Sveti Duh”, and after signing the informed consent document. All procedures have been carried out according to the Declaration of Helsinki. All experimental procedures have been approved by the ethics committee of the Clinical hospital “Sveti Duh”.

Data regarding disease duration, initial and current symptoms (sensory, motor, and brainstem/cerebellar symptoms), number of relapses in the first two years after diagnosis, and total number of relapses (where applicable) were obtained from patients’ medical histories, as well as the annual relapse rate (ARR) and time elapsed from the previous relapse. EDSS scores prior to the current relapse were taken from patients’ histories, with EDSS score 0 assigned to the patients that were diagnosed with MS during their current, first clinical attack. Data on intrathecal immunoglobulin production and presence of oligoclonal bands (OCB) in the CSF were obtained where available. All patients have undergone magnetic resonance imaging (with a magnetic field strength of 1.5 T (Koninklijke Philips N.V., Eindhoven, Netherlands), focusing on the brain and cervical medulla, and including the assessment of lesions after administration of gadolinium. Distribution of lesions was recorded for each patient, as well as the existence of gadolinium-enhancing lesions. The basic characteristics of the study group are presented in Table 1.

After signing the informed consent, 4 mL of venous blood were collected into standard clot activator serum vials. After a 30-min rest at room temperature, serum was separated by centrifuging for 10 min/2000 rpm, and samples were stored at −20 °C. In a fraction of patients (*N* = 11) undergoing a diagnostic lumbar puncture, 0.5 mL of CSF was collected and stored in the same conditions. Appropriate measures were taken to protect confidentiality.

The presence of ANA was assessed using indirect immunofluorescence assay on HEp-2 cells, in standard 1:100 dilution (EUROIMMUN Medizinische Labordiagnostika AG, Lübeck, Germany). In this test, patients’ sera are incubated with slides coated with aforementioned cells. Antibodies directed against cellular antigens, if present, bind to their antigens present in those cells. Afterwards, slides are incubated with FITC (fluorescein isothiocyanate) conjugated antibodies that bind to patient’s antibodies and enable detection under a fluorescent microscope. ANA+ samples were additionally analyzed for the presence of specific autoantibodies (dsDNA, SSA, SSB, Sm, RNP, Scl-70, Jo-1, centromere B and histones) using the AtheNA Multi-Lyte^®^ ANA-II Plus microbead based system (Zeus Scientific, Inc., Branchburg, NJ, USA). This system utilizes polystyrene microbeads coated with antigens to bind to specific antibodies if they are present in a patient’s serum. After this initial incubation, the antibody-bead complex is incubated with PE-conjugated anti-Fc antibodies. The resultant complex is then available for analysis via Luminex^®^ 100/200™/xMAP^®^ tehnology (Luminex Corporation, Austin, TX, USA), in which two laser beams (one for qualitative and other for quantitative analysis) are used to assess the sample.

Levels of circulating sFas and sFasL in sera of patients were determined using the LEGENDplex cytometric bead-based array (BioLegend, San Diego, CA, USA), according to the manufacturer’s instructions. CBA was performed using the LEGENDplex Human CD8/NK Panel (Biolegend, San Jose, CA, USA) to assess the levels of sFas and sFasL in the patients sera and CSF. Undiluted sera and CSF were incubated with capture beads, coated with a capture antibody specific for each soluble protein, and identified by their scatter properties and fluorescence intensity in APC channel. The samples were then washed and incubated with the secondary antibodies conjugated with biotin, and detected by streptavidin-PE. The concentration of the bound analyte was determined by the intensity of the PE fluorescence, depending on the amount of bound PE. The detection reagents were analyzed using Attune flow cytometer (Applied Biosystems, Thermo Fisher Scientific, Waltham, MA, USA). Concentrations of sFas and sFasL were determined using LEGENDplex Data Analysis Software (BioLegend), based on standard curves generated by data acquired from protein standards provided within the kit.

The statistical analysis was performed using MedCalc (version 19.1.6; MedCalc Software Ltd., Ostend, Belgium). Depending on the distribution (determined using the Kolmogorov-Smirnov test for normality) and variable type, data are presented as mean ± SD or median ± IQR. The differences were assessed using the Mann-Whitney test, Student’s *t*-test, or Fisher’s test. Associations between variables were assessed using Spearman’s rank correlation. Statistical significance was set to *p* < 0.05.

All data analyzed during the current study are available in “figshare” repository at https://doi.org/10.6084/m9.figshare.12581582.v1

## 3. Results

**Figure 1:**
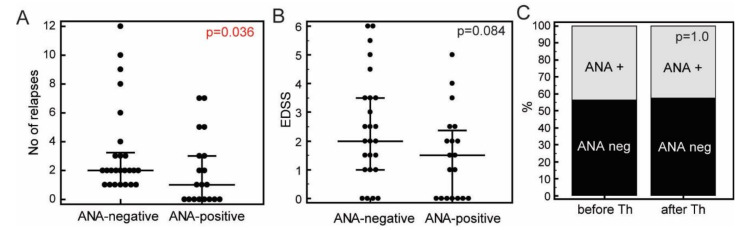
Total number of relapses (**A**) and expanded disability status scale (EDSS, **B**) in ANA-negative (*N* = 25) and ANA-positive (*N* = 19) patients with RRMS, and relative frequencies of ANA in groups of patients whose samples were taken before the initiation of corticosteroid therapy (before Th, *N* = 16), and within 24 h after the initiation of therapy (after Th, *N* = 28, **C**). ANA positivity was determined using HEp-2 IIF test (EUROIMMUN). Markers, individual patients’ values, horizontal lines and bars, median ± IQR; *p* values, Mann-Whitney test (**A**,**B**) or Fisher’s exact test (**C**).

The presence of ANA was found in 19 (43.2%) of all patients. Over the course of disease, ANA positivity tended to decrease, and the most remarkable difference was observed in patients with disease duration of less than one year (8/9, 88.89% of patients were ANA-positive) in comparison with those with disease duration of more than one year (11/35, 31.43%) patients, *p* = 0.003, Fisher’s exact test). Correspondingly, the median number of relapses was significantly lower in ANA positive patients (1, [IQR 0-3] than in ANA-negative patients (2 [IQR 1–3.25], *p* = 0.036, Mann-Whitney test) (Figure 1A). In addition, expanded disability status scale (EDSS) scores were slightly, although non-significantly lower in the ANA-positive (1.5 [IQR 0–2.375]) than in ANA-negative (2.0 [IQR1–3.5], *p* = 0.08) patients (Figure 1B). Annualized relapse rate was not different between groups of ANA-positive (0.50 [IQR 0.23–1.03]) and ANA-negative patients (0.47 [IQR 0.38–1.00], *p* = 0.581). Specific auto-antibodies were detected only in 5/19 (26.32%) ANA-positive patients, and in none of ANA-negative patients. Frequency of ANA-positivity did not differ in relation to age, sex, or other disease characteristics. Initiation of the corticosteroid therapy did not affect the ANA positivity, which was similar in patients whose samples were taken before (7/16, 43.75%) and within 24 h after the initiation of therapy (12/29, 41.38%, *p* = 1.0, Fisher’s exact test, Figure 1C).

**Figure 2:**
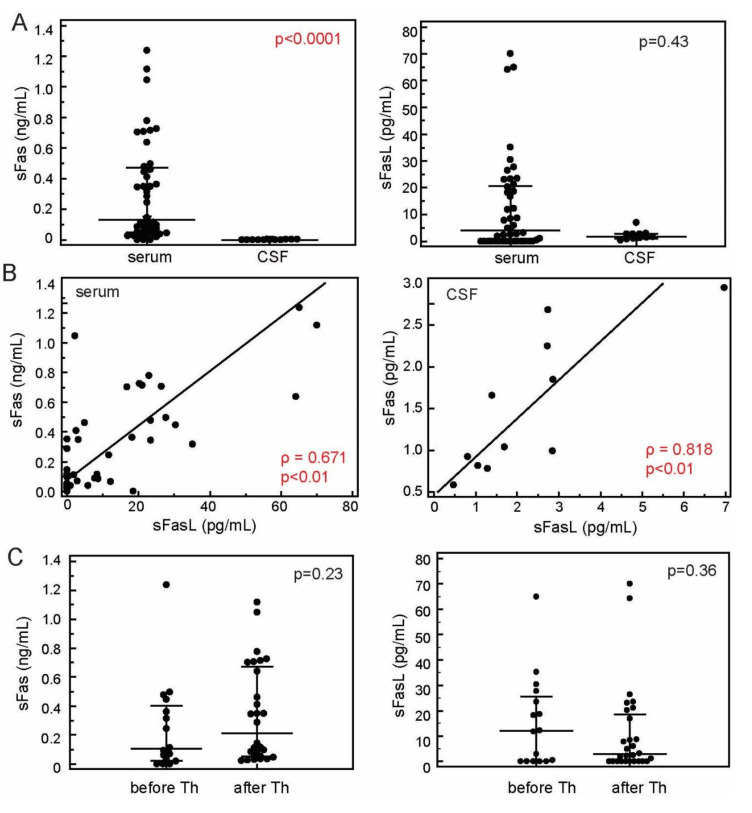
Concentrations of the sFas and sFasL in the sera (*N* = 44) and cerebrospinal (CSF) fluid (*N* = 11) of patients with RRMS (**A**). Association of concentrations of the sFas and sFasL in the sera (*N* = 44) and CSF (*N* = 11) of patients with RRMS (**B**). Concentrations of the sFas and sFasL in the sera of patients with RRMS before the initiation of corticosteroid therapy (before Th, *N* = 16) and within 24 h after initiation of therapy (after Th, *N* = 28) (**C**). Concentrations of sFas and sFasL were measured using cytometric bead-based arrays (LEGENDplex, Biolegend). Markers, individual patients’ values, horizontal lines and bars, median ± IQR; *p* values, Mann-Whitney U-test (**A**,**C**), Spearman’s rank correlation (**B**), ρ, correlation coefficient.

Concentrations of sFas in patients’ sera were significantly higher (130.21 [IQR 48.02–468.95] pg/mL) in comparison to concentrations of sFasL (4.06 [IQR 0.00–20.66] pg/mL, *p* < 0.0001, Mann-Whitney test). Concentrations of sFas (1.50 [IQR 0.84–2.15] pg/mL) and sFasL (1.69, [IQR 1.12–2.82] pg/mL) were similar in the CSF (*p* = 0.24, Mann-Whitney test). The serum concentration of sFas was significantly higher than its concentration in the CSF (*p* < 0.0001, Mann-Whitney test), whereas the concentrations of sFasL were similar in both serum and the CSF (*p* = 0.43, Mann-Whitney test, Figure 2A). There were no differences in sFas and sFasL levels when patients were stratified according to sex, age, and duration of the disease, although the sFasL tended to be lower in patients with longer disease duration (15.31 ± 19.47 vs. 4.75 ± 8.96 pg/mL in patients whose disease lasted 0–9 years vs. 10 years or more, respectively, *p* = 0.09, *t*-test). Despite differences in the levels, concentration of sFas was strongly associated with the concentration of FasL in both serum (ρ = 0.671, *p* < 0.0001) and CSF (ρ = 0.818; *p* = 0.002, Figure 2B) pointing to their interdependence on both peripheral and CNS levels. There was no association between serum and CSF concentrations of either sFas (ρ = 0.0182; *p* = 0.9577) or sFasL (ρ = −0.239; *p* = 0.4800). Concentrations of sFas and sFasL did not differ significantly when measured before initiation of corticosteroid therapy (sFas, 104.12 [IQR 24.48–403.56] pg/mL; sFasL, 12.01 [IQR 0.00–25.58] pg/mL]) and within 24 h after the initiation of therapy (sFas, 215.20, [IQR 52.64–670.25] pg/mL, *p* = 0.37; sFasL, 2.86 [IQR 0.00–5.76] pg/mL], *p* = 0.46, Figure 2C).

**Figure 3:**
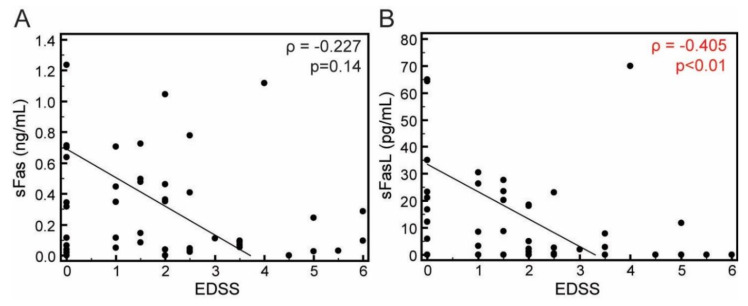
Association of concentrations of the sFas (**A**) and sFasL (**B**) in the sera of patients with RRMS with their EDSS score (*N* = 44). Concentrations of sFas and sFasL were measured using cytometric bead-based arrays (LEGENDplex, Biolegend). Markers represent individual patients’ values; *p* values, Spearmans rank correlation ρ, correlation coefficient.

The serum concentrations of sFas appear to be weakly inversely associated with EDSS score assessed in the previous remission, although this association was not statistically significant (ρ = −0.227, *p* = 0.14, Figure 3A). A significant negative association was established for sFasL (ρ= −0.405, *p* = 0.006, Figure 3B), pointing to a conclusion that lowered peripheral concentrations of both mediators during relapse would indicate higher level of accumulated disability. No association to EDSS score was established for CSF concentrations of both mediators.

**Figure 4:**
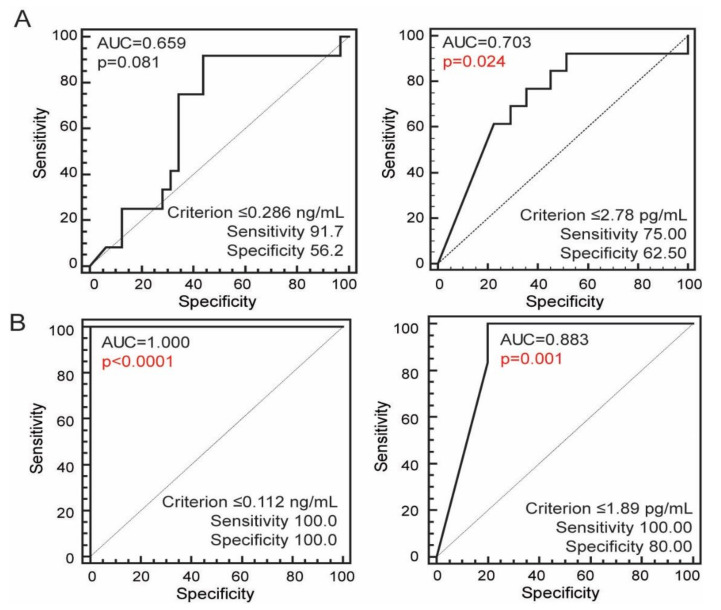
Ability of sFas and sFasL concentrations in sera of all patients with RRMS (**A**, *N* = 44), and patients with disease duration ≥ 10 years (**B**, *N* = 11) to discriminate between RRMS patients with mild or absent disability (EDSS score ≤ 3.0) and patients with moderate to severe disability (EDSS > 3) [36]. The sensitivity, specificity, and a “cut off” point (criterion) of sFas and sFas concentrations were assessed by ROC curve analysis. AUC, area under curve; ROC, receiver operating characteristics.

To assess the ability of serum concentrations of both soluble factors to discriminate the patients with higher from patients with lower level of disability, we divided patients according to their EDSS score in two groups: EDSS ≤ 3.0 (absent to mild disability, *N* = 32) and EDSS > 3. (moderate to severe disability, *N* = 12) [37], and performed ROC curve analysis. We found that lowered concentrations of sFasL measured in the relapse indicated more severe outcomes of disease (Figure 4A). Although EDSS worsens progressively during disease (ρ = 0.667, *p* < 0.0001), the concentrations of both soluble factors were not associated with the disease duration alone (sFas ρ = −0.040, *p* = 0.80; sFasL −0.276, *p* = 0.07), and ROC curve analysis in patients grouped by disease duration (applying thresholds of 2, 5, and 10 years to group the patients) could not determine a significant cutoff values of sFas nor the sFasL to segregate patients. However, the concentrations of both sFas and sFasL below the cutoff value were the best discriminators of higher EDSS scores, and more severe degrees of disability in a group of patients with disease duration >10 years (Figure 4B). In addition to EDSS score, concentration of sFasL was also inversely associated with the number of experienced relapses (ρ = −0.307, *p* = 0.042), while such association was not established for sFas. Other patients’ characteristics such as the number of relapses in the first two years, ARR, and time elapsed from previous relapse were not apparently associated with concentrations of soluble apoptosis markers, nor were sFas and sFasL different in subgroups of patients divided by initial presentation, current symptoms, localization of demyelinating lesions or the presence of active lesions.

**Figure 5:**
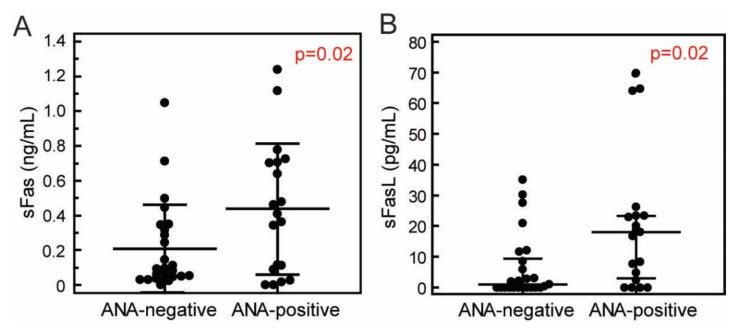
Concentrations of the sFas (**A**) and sFasL (**B**) in the sera of ANA-positive (*N* = 19) and ANA-negative (*N* = 25) patients with RRMS. Concentrations were measured using cytometric bead-based arrays (LEGENDplex, Biolegend). ANA positivity was determined using HEp-2 IIF test (EUROIMMUN). Markers, individual patients’ values, horizontal lines and bars, mean ± SD (**A**) or median ± IQR (**B**); *p* values, *t*-test (**A**) or Mann-Whitney test (**B**).

The concentrations of sFas were higher in ANA positive (437.83 ± 376.79 pg/mL), in comparison to ANA-negative group of patients (208.67 ± 251.94 pg/mL, *p* = 0.02, *t*-test). Patients with presence of ANA also had significantly higher concentrations of sFasL, (18.20 [IQR 3.12–23.45] pg/mL) in comparison to ANA-negative patients (1.00 [IQR 0.00–9.44] pg/mL, *p* = 0.02, Mann-Whitney test, Figure 5).

## 4. Discussion

According to our study, ANA were generally present in less than half of RRMS patients, and their frequency is especially common early in the disease, which is also reflected in significantly lower number of relapses and tendency towards lower EDSS scores in the group of ANA-positive patients. ANA in MS rarely belong to the specific groups of autoantibodies related to diseases from the SARD spectrum, pointing to their different specificities, which yet need to be determined. ANA-positivity in early stages of the disease could be a result of enhanced peripheral apoptosis of autoreactive lymphocytes, as an attempt to regulate the autoimmune processes, thus exposing lymphocyte intracellular antigens, and eliciting autoantibody production. A relative change in the abundance of nuclear antigens, with fewer epitopes presented to the immune system [38], could account for a larger proportion of ANA negative patients later in the course of the disease. This explanation would be in concordance with the current knowledge about the pathogenic mechanisms of ANA production in other autoimmune diseases, particularly of SARD spectrum of diseases, where ANA production in autoimmune diseases is thought to be a result of excessive apoptosis of cytotoxic lymphocytes [21,22], and a reduction in those events could remove the substrate for their production.

In addition, serum concentrations of sFas/sFasL do not seem to reflect the ongoing events within the CNS, judging by the poor correlation between serum and CNS levels of those apoptosis mediators, and their generally low concentrations in CNS. Strong association between concentrations of sFas and sFasL points to their co-dependency in immune regulation in MS. To our knowledge, other studies of those apoptosis molecules in patients with MS did not report on their association with one another [39]. This phenomenon was observed earlier only in patients with SLE or rheumatoid arthritis, but not in other rheumatic diseases [40]. Strong association between those molecules was reported in some other immune-mediated diseases, such as immune thrombocytopenic purpura [41], while their inverse association was reported in patients with a non-immune mediated acute pancreatitis [42], which could imply differences in apoptotic regulation depending on the involved components of the immune system. Inverse association of concentrations of soluble apoptotic molecules measured in relapse with EDSS score in previous remission may indicate inadequate removal of autoreactive lymphocytes and thus limited attenuation of the autoreactive processes during the relapse, which results in increased immune damage, and greater accumulation of disability in time. This is supported by the fact that both soluble mediators better discriminate patients with moderate or severe disability, from patients with mild or absent disability in a group of patients with long (>10 years) of disease duration. It has been demonstrated that sFas concentrations are also associated by higher activity of SLE [43], and juvenile SLE assessed by SLEDAI (Systemic Lupus Erythematosus Disease Activity Index) score [44], and that higher concentrations of sFas are associated with higher percentages of activated T and NK lymphocytes [41]. Conversely, their levels in SLE diminish following steroid therapy [40]. The lack of differences in sFas and sFasL concentrations of our patients regarding steroid therapy was possibly due to the fact that such therapy was never started more than a day before sampling, before they could exert their full effect.

Potential co-dependence of concentrations of soluble apoptotic mediators and ANA is reflected by their higher circulating concentrations in the groups of patients with ANA positivity, which might reflect a higher activity of the apoptotic pathways, as an effort to eliminate the autoreactive lymphocytes from the circulation. This result needs to be interpreted with caution due to the insufficiently explained and seemingly ambiguous role of these soluble variants of membrane-bound proteins.

The possible limitations in extrapolating the results of this study to the general MS population arises from the fact that due to exclusion of patients using DMT, there was possibly a tendency towards the inclusion of patients with a milder disease course, aggravated by the fact that the policies of the relevant insurance company during the recruitment period precluded the prescription of DMT to the patients that have not met the somewhat stringent criteria for disease activity. Additional limitation is a small number of CSF samples, which was a result of lack of indication for lumbar puncture in our study group. Concentrations of soluble apopototic factors in CSF were therefore not used to draw conclusions on their association with disease parameters, but we found it informative to include the data on relative abundance of sFas on the peripheral in comparison to CNS level, as well as co-dependence of sFas and sFasL on both CNS and peripheral levels, which could be derived despite the small sample size.

This study provides novel data on the linkage between the antinuclear antibodies, soluble apoptotic molecules, and certain disease characteristics in MS, opposing the consideration of ANA as pure coincidence or a false positive result. Further studies are required to provide the mechanistic explanation of this phenomenon, to better understand immune regulatory and apoptotic processes in the context of autoimmunity.

## Figures and Tables

**Table 1:** Characteristics of the study group.

Age (years, male/female)	38.43 ± 10.25 (33.91 ± 9.72/40.12 ± 10.07)	*N* = 44
Disease duration (years, male/female)	6 [1–10](5.5 [1.5–7.5]/6.5 [1–13]) *	*N* = 44
Number of experienced relapses (male/female)	3 [2–4](3.5 [2–5]/3 [1.5–4.0]) *	*N* = 44
Number of relapses in first two years of the disease (male/female)	1 [1–2](2 [1.0–2.5]/1 [1–2]) *	*N* = 32
Annualized relapse rate (male/female)	0.47 [0.33–1.00] (0.75 [0.44–1.25]/0.42 [0.28–0.71]) *	*N* = 35
EDSS (male/female)	2 [0.50–3.25] (2.0 [1.25–3.00]/1.75 [0.00–3.25]) *	*N* = 44

Data are presented as mean ± SD or median [IQR] *.
